# Association of *IL28B* SNPs rs12979860 and rs8099917 on Hepatitis C Virus-RNA Status in Donors/Recipients of Living Donor Liver Transplantation

**DOI:** 10.1371/journal.pone.0156846

**Published:** 2016-06-08

**Authors:** King-Wah Chiu, Toshiaki Nakano, Kuang-Den Chen, Chih-Che Lin, Tsung-Hui Hu, Shigeru Goto, Chao-Long Chen

**Affiliations:** 1 Division of Hepato-Gastroenterology, Department of Internal Medicine, Kaohsiung Chang Gung Memorial Hospital, Kaohsiung, Taiwan; 2 Department of General Surgery, Kaohsiung Chang Gung Memorial Hospital, Kaohsiung, Taiwan; 3 Liver Transplantation Program, Kaohsiung Chang Gung Memorial Hospital, Kaohsiung, Taiwan; 4 Chang Gung University College of Medicine, Taoyuan, Taiwan; The University of Hong Kong, HONG KONG

## Abstract

To investigate the effect of *IL28B* single nucleotide polymorphisms (SNPs) (rs8099917 and rs12979860) in the donors and recipients on the outcome of Hepatitis C virus-RNA clearance after living donor liver transplantation (LDLT). The rs8099917 and rs12979860 genotypes in 50 donor and recipients pairs were explored on the pre-operative day (POD) and post-operative day 30 (POD30). There was a significant difference in HCV-RNA clearance before (12%, 6/50) and after (48%, 24/50) liver transplantation (P < 0.001). The rs8099917 genotype TT was dominant in both the recipients (82%, 41/50) and donors (86%, 43/50), but had no significant effect on HCV-RNA clearance (87.5%, 21/24) and recurrence (76.9%, 20/26) after LDLT. One recipient was detected with genotype GG on POD, which changed to genotype GT on POD30. Prevalence of rs12979860 genotype CT was 98% (49/50 recipient) and 92% (46/50 donor) and prevalence of genotype CC was 2% (1/50 recipient) and 8% (4/50 donor) on POD and POD30, respectively. Of the 4 recipients with rs12979860 genotype CC on POD30, 3 recipients (12.5%, 3/24) exhibited HCV clearance and 1 experienced recurrence (3.9%, 1/26), however, this was not statistically significant. In conclusion, alterations in *IL28B* SNP genotype may occur after LDLT, leading to modifications in the host genome or donor proteome by HCV. This predicted mechanism will need to be investigated further.

## Introduction

Complication arising due to hepatitis C virus (HCV) infection along with liver cirrhosis and hepatocellular carcinoma is an important public health issue worldwide [1]. A major challenge in combating HCV infection is the prevention of HCV recurrence following living donor liver transplantation (LDLT) [2]. The immune system of the host plays a very important and preventative role against HCV recurrence [3]. If a patient is exposed to HCV, resulting in liver infection, then it could be assumed there is a defect in the host immunity. Therefore, it is of interest to study what immune factors within the hepatocytes have been modified by the healthy donor liver graft following LDLT that results in HCV clearance. In our previous study, cytochrome p450 isoenzyme genotypes presented a homogenous modification phenomenon following LDLT, with different genomic characteristics between the recipients and their donors [4–6]. HCV targets liver hepatocytes, whilst the hepatocytes themselves express interleukins, encoded by the *IL28B* gene. *IL28B* single nucleotide polymorphisms (SNP), rs8099917 and rs12979860, have been reported to play a major role in the sustained viral response for interferon plus ribavirin therapy [7–9]. We recently observed that this kind of *IL28B* with favorable genotypes may contribute to spontaneous clearance of HCV infection [10]. Therefore, in the present study, we aimed to explore the *IL28B* SNP of living donors and their recipients before and after LDLT in relation to HCV clearance or recurrence, as a part of the liver transplantation program.

## Methods

### Patients

Between April 2015 and March 2016, 50 pairs of recipients and their donors whom had undergone LDLT were enrolled in this prospective cohort study. Of the 50 recipients, 22 were male and 28 were female with a mean age of 59.14 years (ranged 48 to 72). Of the 50 donors, 26 were male and 24 were female with a mean age of 34.5 years (ranged 20 to 54). All of the 50 recipients reacted positive to anti-HCV antibody, with 5 of the patients presenting with hepatocellular carcinoma. Of these patients, 33 harbored HCV genotype 1; 4 had genotype 1a and 29 had genotype 1b. Another 11 patients were with HCV non-genotype 1; 7 with genotype 2a, 3 with genotype 2b and 1 with genotype 3b. The HCV genotype was uncertain in remaining 6 recipients due to undetectable HCV-RNA before LDLT. In our liver transplantation program, there were 5 anti-HCV management strategies.^2^ No anti-viral therapy was administered to recipients with a history of interferon(IFN)/ribavirin(RBV) treatment or who were HCV-RNA negative prior to LDLT. A fixed dosage of Peg-IFN-α2a (135 μg/week) plus RBV (10 mg/kg per day) was administered for 4 weeks to the HCV-RNA positive recipients who crossed the 4-week waiting period, in accordance with clinical safety considerations that have previously been reported.^2^ In cases of acute, chronic HCV with liver failure, post-transplant fixed dosage pegIFN-alpha/RBV treatment was administered to HCV-RNA positive recipients. Since April 2015, Direct-Acting Antiviral Agents (DAA) have been available in our program. A 3-month post-transplant DAA treatment using Harvoni (Sofosbuvir 400 mg plus Ledipasvir 90 mg)/tab (one tab per day), in addition to RBV (400 mg per day) was administered to 5 recipients who presented with high HCV-RNA levels following LDLT. Because the DAA were not covered by government insurance, a fixed dosage pegIFN-alpha/RBV treatment was administered to the other HCV-RNA positive residual recipients after LDLT for 6 months.

### IL28B polymorphisms

Genomic DNA was extracted from the peripheral blood mononuclear cells of LDLT donors and recipients on pre-operative day (POD) and post-operative day 30 (POD30) using the QIAamp DNA Blood Mini Kit (Qiagen, Hilden, Germany). Genotyping of *IL28B* polymorphisms was performed using ABI Custom TaqMan SNP Genotyping Assays and allelic discrimination kit (Applied Biosystems, Foster City, CA, USA). The real-time PCR reactions were performed in 96-well microplates, using the ABI 7500 Fast Real-Time Polymerase Chain Reaction System (Applied Biosystems International, Framingham, MA) in accordance with the manufacturer's instructions. The *IL28B* SNP rs8099917 was defined as TT, GT or GG genotype; and rs12979860 was defined as CC, CT or TT genotype, as recommended by the manufacturer. All genotypes of *IL28B* SNP rs8099917 and rs12989860 were assayed in duplicate in order to assess inter-assay precision. Patients with HBsAg positive serum or pediatric transplantation were excluded from this study.

### Quantification of HCV-RNA and HCV Genotyping

HCV-RNA was determined by a standardized reverse-transcription polymerase chain reaction assay (Amplicor; Roche Diagnostics, RotKreuz, Switzerland), using biotinylated primers for the 5’ noncoding region following the manufacturer’s instructions. The lowest detection limit of this assay was 15 IU/mL.

HCV genotype was determined using a reverse hybridization assay (Inno-LiPATM HCV II; Innogenetics N.V., Gent, Belgium) and HCV-Amplicor products. HCV serotypes 1 and non-1 correspond to genotypes 1a/1b, 2a/2b and 3b, respectively.

### Ethics statement

Written informed consent was obtained from each patient that participated in this study. The study protocol conformed to the ethical guidelines of the Declaration of Helsinki and was approved by the ethics review committee of Chang Gung Memorial Hospital (No: 103-0679C). None of the transplant donors were from a vulnerable population, and written informed consent was provided by all donors, or their next of kin.

### Statistical analysis

Data were analyzed using the Statistical Package for repeated measures (SPSS v. 22.0 for Windows, IBM Corp., Armonk, NY, USA). A Student’s *t*-test was used to compare the quantitative HCV-RNA in clearance and recurrence following LDLT. Fisher’s Exact test was used to compare the difference in *IL28B* SNP rs8099917 and rs12979860 genotypes in the recipients and their donors. Differences were considered statistically significant when P < 0.05.

## Results

### Evaluation of the efficacy of HCV-RNA status in recipients with LDLT

Of the 50 HCV positive patients who received LDLT, serum HCV-RNA was undetectable in 48% (24/50) and detectable in 52% (26/50) following the procedure. There was a significant difference (P = 0.001) in the rate of undetectable serum HCV-RNA before and after LDLT (12%, 6/50 vs 48%, 24/50) (Table 1). According to the serum viral load, both HCV genotype 1 and genotype non-1 were significantly responsible (P = 0.000 and P = 0.006) for LDLT with optimal management before and after LDLT (75%, 33/44 vs 47.7%, 21/44; 25%, 11/44 vs 11.4%, 5/44, respectively) (Table 1).

**Table 1:** Genotypes, serum HCV-RNA and viral loads of hepatitis C virus in the recipients before and after living donor liver transplantation (LDLT).

Category	Before LDLT	After LDLT	P value
**Genotype**			
*G*1	33 (75.0)	21 (47.7)	0.000^a^
*G*1a	4	2	
*G*1b	29	19	
*G* non-1	11 (25.0)	5 (11.4)	0.006^b^
*G*2a	7	2	
*G*2b	3	1	
*G*3b	1	1	
uncertain	6	6	
**Serum HCV-RNA**				
Detectable (%)	44 (88)	26 (52)	0.000^c^
Undetectable (%)	6 (12)	24 (48)	
**Viral load (IU/ml)**				
Minimum	670	103	
Maximum	28700000	20550304	
Total, mean	14350335	10275203.5	0.000*
SD	20293490.86	14531186.48	
*G*1, mean	14351158.00	1483281.50	0.01
SD	20292326.96	1818629.85	
*G*1a, mean	2645689.50	9604623.00	>0.05
SD	3612340.55	9666681.44	
*G*1b, mean	5901158.00	10373810.00	0.03
SD	8342222.359	14391735.83	
*G* non-1, mean	3087626.50	96022.50	>0.05
SD	4365615.75	135650.66	

^a, b, c^ = Fisher’s Exact Test

* = Wilcoxon Signed Ranks Test (2-tiled); *G* = genotype; SD = standard deviation

### Comparison of the genotypes of IL28B between recipients and donors

Up to 86% (43/50) of donors expressed rs8099917 genotype TT, as well as 82% (41/50) of the recipients (Table 2). There was not only no statistical significance in the predominant expression between donors and recipients (86% vs 82%, NS), but also between the HCV-RNA clearance and recurrence (87.5%, 21/24 vs 76.9%, 20/26) following LDLT (Tables 1 and 2). In the donors, there was limited expression of genotype GT, at 8% (4/50) and of genotype GG, at 6% (3/50). In the recipients, genotype GT was 16% (8/50) and genotype GG was 2% (1/50) on POD (Table 2). Incidentally, rs8099917 genotype GG was converted to genotype GT, leading to 18% (9/50) expression in the recipients on POD30 (Table 2). This biogenetic alteration was also found in the rs12989860 genotype CC, which expressed in 2% (1/50) of recipients on POD and 8% (4/50) on POD30. Genotype CT was expressed in 98% of recipients (49/50) on POD and 92% (46/50) on POD30, but there was no statistical significance between them (Table 2). All of the genotypes were assayed in duplicate for final confirmation. Regarding the impact of HCV-RNA status, no statistical significance was observed for rs8099917 donor genotype TT (86%, 43/50) and recipient genotype TT (82%, 41/50) between the HCV-RNA clearance (87.5%, 21/24) and HCV-RNA recurrence (76.9%, 20/26) rate at POD30 (Table 2). Similar statistically insignificant results were observed for rs12989860 donor genotype CT (86%, 43/50) and recipient genotype CT (92%, 46/50) between the HCV-RNA clearance (77.5%, 21/24) and HCV-RNA recurrence (96.1%, 25/26) rate at POD30 (Table 2).

**Table 2:** *IL28B* single nucleotide polymorphism genotypes of rs8099917 and rs12979860 in the donors and recipients compared to the HCV-RNA status of recipients before (POD) and after (POD30) living donor liver transplantation.

Category	Donors (n = 50)			Recipients (n = 50)				P value
Age (mean) years	20~54 (34.5)			48~72 (59.14)				0.000
Gender, Male/Female	26/24			22/28				>0.05
HCV-RNA status of recipients after LDLT		Clearance	Recurrence	Clearance	Recurrence	LDLT		
		n = 24	n = 26	n = 24	n = 26	POD	POD30	
rs8099917								
TT (%)	43 (86)	22 (97.7)	21 (80.8)	21 (87.5)	20 (76.9)	41 (82)	41 (82)	>0.05
GT (%)	4 (8)	1 (4.2)	3 (11.5)	3 (12.5)	6 (23.1)	8 (16)	9 (18)	>0.05
GG (%)	3 (6)	1 (4.2)	2 (7.7)	0	0	1 (2)	0	>0.05
rs12979860								
CC (%)	7 (14)	2 (8.3)	5 (19.2)	3 (12.5)	1 (3.9)	1 (2)	4 (8)	>0.05
CT (%)	43 (86)	22 (91.7)	21 (80.8)	21 (77.5)	25 (96.1)	49 (98)	46 (92)	>0.05
TT (%)	0	0	0	0	0	0	0	

LDLT = living donor liver transplantation; POD = pre-operative day; POD30 = post-operative day 30

### Comparison of the Anti-viral strategy between IL28B and HCV-RNA clearance after LDLT

**Fig 1:**
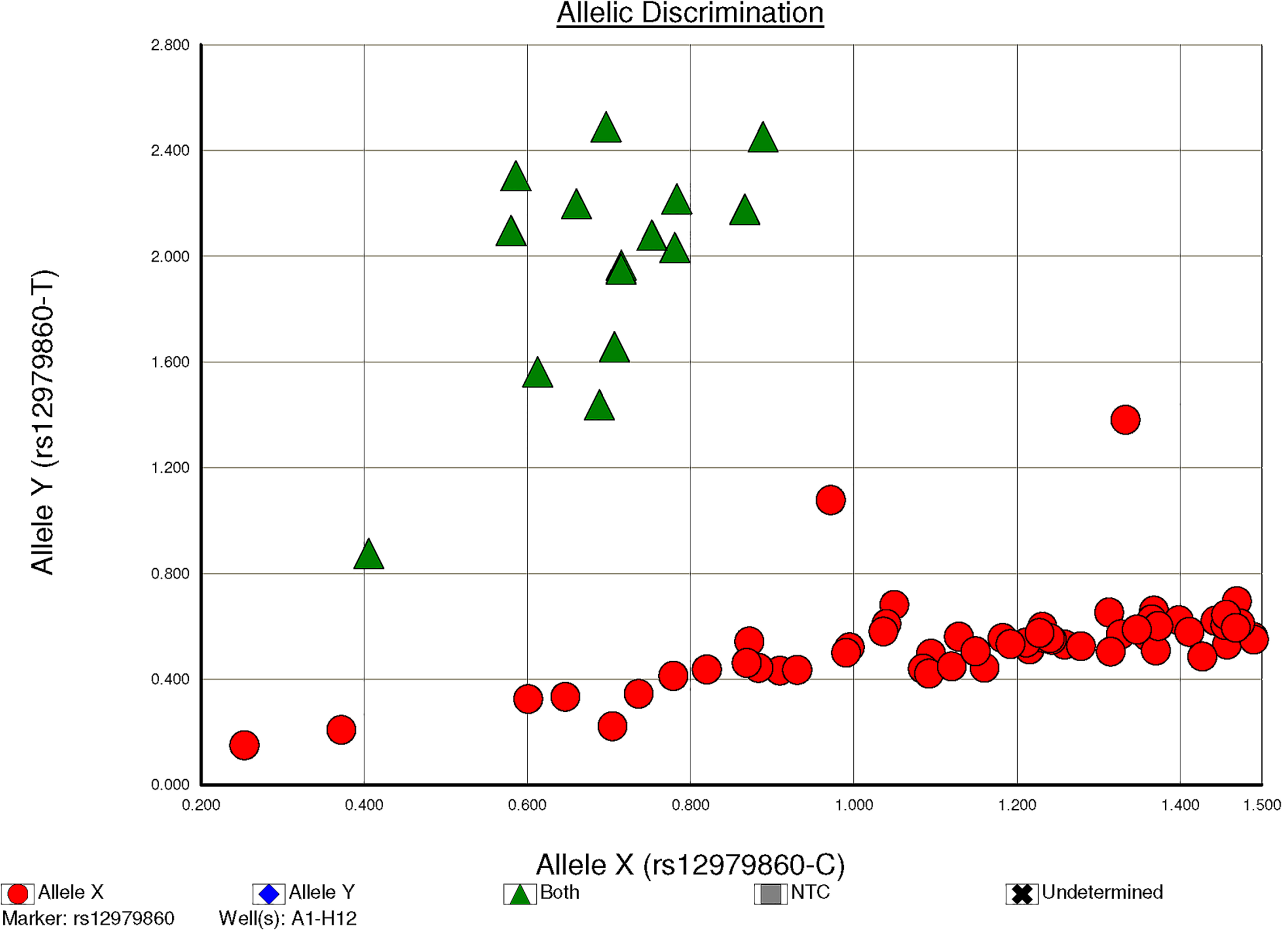
*IL28B* polymorphism rs12979860 allelic discrimination with 96-well microplates interpreted using 7500 Fast Real-Time Polymerase Chain Reaction System (Applied Biosystems International, Framingham, MA). Red ball represented genotype CC. Green triangle represented genotype CT.

Among the 24 recipients who showed HCV-RNA clearance, 4 had no treatment before LDLT, 5 had previously received pegIFN/RBV for 6 to 12 months, 4 had been treated with pre-transplant fixed dosage pegIFN/RBV for 4 months, 6 received post-transplant fixed dosage pefIFN.RBV for 6 months, and 5 received DAA for 3 months, after 90 days of LDLT. The DAA regiment was a 3-month course of Harvoni (Sofosbuvir 400 mg plus Ledipasvir 90mg)/tab (one tab per day) administered in addition to RBV (400 mg per day) (Table 3). There was no evidence of a relationship between the *IL28B* SNP genotypes and different anti-viral strategies for HCV-RNA clearance efficacy. All of the optimal anti-viral strategies were beneficial for HCV-RNA clearance in patients that underwent LDLT. For the rs8099917 genotype, there is no evidence that the donor affects the recipient’s *IL28B* SNP after LDLT. There was no difference in TT and GT genotype composition between the POD and POD30 in any of the different anti-viral treatment strategies. In contrast, for rs12979860, there were 5 recipients expressing genotype CT at the POD. Two of these 5 recipients had been altered to genotype CC (Fig 1) at POD30, prior to DAA treatment (Table 3). It should be emphasized that DAA was given 3 to 6 months after LDLT. Blood sampling of POD30 was performed 30 days following LDLT.

**Table 3:** *IL28B* single nucleotide polymorphism (SNP) genotypes of rs8099917 and rs12979860 in 24 recipients with HCV-RNA clearance after living donor liver transplantation and their anti-viral treatment strategy.

Category	rs8099917			rs12979860		
	Donor	Recipient		Donor	Recipient	
		POD	D30		POD	D30
No treatment, n = 4	4TT	4TT	4TT	1CC, 3CT	4CT	4CT
History of pegIFN/RBV, n = 5	4TT, 1GG	4TT, 1GT	4TT, 1GT	1CC, 4CT	5CT	5CT
Pre-transplant pegIFN/RBV, n = 4	3TT, 1GT	4TT	4TT,	4CT	4CT	4CT
Post-transplant pegIFN/RBV, n = 6	6TT	6TT	6TT	6CT	1CC, 5CT	1CC, 5CT
DAA, n = 5	5TT	3TT, 2GT	3TT, 2GT	5CT	5CT	2CC, 3CT

pegIFN/RBV = fixed dosage pegIFN-alpha (135 μg/week) plus ribavirin (10 mg/kg/day); pre-transplant = 4 months course before liver transplant; post-transplant = 6 months course after liver transplant; DAA = direct-acting antiviral agent

## Discussion

The *IL28B* gene and its polymorphisms are strongly associated with spontaneous clearance and anti-viral treatment response in patients with chronic HCV infection [8]. The two important SNPs, rs8099917 and rs12979860, have been reported to have a close association with the sustained viral response in the Asian population [7,9]. Because the liver biopsy was not available after LDLT in this study, we examined the peripheral blood at different periods to compare the difference between before (POD) and after (POD30) LDLT. To reduce the impact of unstable factors, such as acute rejection following LDLT, we used a 30-day term following liver transplantation as an index [11–12]. In the current study, the favorable rs8099917 genotype TT had a majority distribution amongst the recipients and their donors, 82 to 86%, respectively. Basically, the donor liver graft was implanted in the recipient, but the original genotype of the recipient remained unchanged by the donor graft in the peripheral blood after LDLT [13]. despite a difference in the genotypes TT, TG and GG between the donors and recipients before LDLT. Although high expression of the favorable genotype TT in the recipients and their donors led to a 48% HCV-RNA clearance rate, it was difficult to confirm the role of donor genotype due to a 52% HCV-RNA recurrence rate in genotype TT following LDLT.

In the *IL28B* SNP rs12979860, high expression of unfavorable genotype CT in both recipient and donor led to high HCV-RNA recurrence after LDLT. Therefore, *IL28B* polymorphism rs12979860 should be a more important genetic factor in the outcome of HCV infection and treatment [14–15]. In contrast to *CYP2C19*, *CYP3A4*, *CYP3A5* and *MDR1-3536*, the homogenous phenomenon [4–6] seems to be less responsible in the *IL28B* SNP donor modification. Surprisingly, the favorable genotype CC has been altering from the unfavorable genotype CT in recipients following LDLT. The likeliest possibility for this phenomenon comes from the improvement of host immunity after LDLT and secondly, by HCV itself. Structural mutation in the non-structural protein of HCV has been reported to alter the cyclosporine response after liver transplantation [16]. However, polymorphisms alter to predict the response to metastatic breast cancer chemotherapy [17] and tumor necrosis factor, α-induced protein 3 (TNFAIP3) gene mutation in oral squamous cell carcinoma [18]. Therefore, the recipient may possess the ability to alter the genotypes from CT to CC, leading to clearance of HCV-RNA after liver transplantation. In fact, their donors also expressed the same genotype CT in peripheral blood genotyping of the two recipients. Therefore, identifying genotype CC alteration is difficult within the current study. This idea has not appeared previously in the literature. Based on current observations, this novel discovery is worth further investigation.

### Limitation

The major limitation of the current study concerns potential donor selection. Originally, we intended to explore the *IL28B* SNP rs12979860 favorable genotype CC from the donor, which may affect the unfavorable genotype TT from the recipient after LDLT. Unfortunately, there was only one case out of 50 donors in whom genotype CC of *IL28B* SNP rs12979860 was expressed. It is therefore difficult to identify this phenomenon in the current study. Meanwhile, the presentation of favorable genotype TT and unfavorable GG genotypes of *IL28B* SNP rs8099917 was distributed similarly in both the recipients and their donors. Therefore, it is not possible to investigate the biogenetic modification between recipients and donors after LDLT.

The current study is limited by the both of the one year-budget and the patient selection of this specific cohort with HCV related LDLT. Clinically, we will continuous to follow up this phenomenon in our liver transplantation program on our on-going liver biopsy study of the liver graft in patients with HCV related LDLT. It should be overcome this limitation and possibility of increasing sample size in the future. Laser capture microdissection of the hepatocyte from the liver graft biopsy on POD30 will be one the best method to identify the finally DNA come from.

In conclusion, the association of the *IL28B* polymorphism genotypes rs8099917 and rs12979860 may play a major role in HCV-RNA clearance, but independent of the donor’s genotype. The rs12979860 genotype may be altered from CT to CC following LDLT, but this will require further investigation.

## Abbreviations

DAA: direct-acting anti-viral agent
HCV-RNA: hepatitis C virus-ribonucleic acid
INF: interferon
*IL28B*: interleukin-28B
LDLT: living donor liver transplantation
RBV: ribavirin
SNP: single nucleotide polymorphism
